# Incomplete recovery of the CD4+/CD8+ ratio is associated with the late introduction of antiretroviral therapy among people living with HIV infection

**DOI:** 10.1590/S1678-9946202466007

**Published:** 2024-02-05

**Authors:** Gabriela da Silva Prates, Mariana Amelia Monteiro, Éricka Constantinov Oliveira, Najara Ataide de Lima Nascimento, Ana Paula Rocha Veiga, Mauricio Domingues Ferreira, Thales José Bueno Polis, Gabriela Prandi Caetano, Beatriz Rodrigues Pellegrina Soares, Marcello Mihailenko Chaves Magri, Luisa Oliveira Pereira, Luiz Augusto Marcondes Fonseca, Wagner Silva Alves, Alberto José da Silva Duarte, Jorge Simão do Rosário Casseb

**Affiliations:** 1 Universidade de São Paulo, Faculdade de Medicina, Instituto de Medicina Tropical de São Paulo, São Paulo, São Paulo, Brazil; 2 Universidade de São Paulo, Hospital das Clínicas, Departamento de Dermatologia, Ambulatório de Imunodeficiências Secundárias, São Paulo, São Paulo, Brazil

**Keywords:** Human immunodeficiency virus, HIV-1, Early therapy, Antiretroviral therapy, cART, Non-HIV-associated diseases, CD4^+^/CD8^+^ ratio

## Abstract

Despite being subject to lower AIDS-related mortality rates and having a higher life expectancy, patients with HIV are more prone to develop non-AIDS events. A low CD4+/CD8+ ratio during antiretroviral therapy identifies people with heightened immune senescence and increased risk of mortality. In clinical practice, finding determinants of a low CD4+/CD8+ ratio may be useful for identifying patients who require close monitoring due to an increased risk of comorbidities and death. We performed a prospective study on the evolution of the CD4^+^/CD8^+^ ratio in 60 patients infected with HIV (80% males), who were subjected to two different antiretroviral regimens: early and deferred therapy. The initial CD4^+^/CD8^+^ ratio was ≤1 for 70% of the patients in both groups. Older age, CD4^+^ cell count at inclusion, Nadir CD8^+^T-cell count, and Initial CD4^+^/CD8^+^ ratio ≤ 1 were risk factors for lack of ratio recovery. In the multivariate analysis, a CD4^+^/CD8^+^ ratio > 1 at the start of the treatment was found to be a determinant factor in maintaining a CD4^+^/CD8^+^ ratio > 1. The nadir CD4^+^T-cell count was lower in the deferred therapy group (p=0.004), and the last CD4^+^/CD8^+^ ratio ≤1 was not associated with comorbidities. Ratio recovery was not associated with the duration of HIV infection, time without therapy, or absence of AIDS incidence. A greater improvement was observed in patients treated early (p=0.003). In contrast, the slope of increase was slower in patients who deferred treatment. In conclusion, the increase in the CD4+/CD8+ ratio occurred mostly for patients undergoing early strategy treatment and its extension did not seem to be related to previous HIV-related factors.

## INTRODUCTION

The CD4/CD8 T-cell ratio is an important indicator of the severity of HIV disease and the response to antiretroviral therapy (ART)^1^. People with HIV (PWH) with persistent CD4/CD8 T-cell ratio inversion (defined as a ratio <1.0) show elevated biomarkers of T-cell activation, exhaustion, and immunosenescence^2^. Since 1997, the Brazilian national AIDS program has provided free antiretroviral therapy (cART) for individuals infected with HIV, initially for patients with a CD4^+^ T cell count below 350 cell/mm^3^ and/or with opportunistic diseases. Since 2015, cART initiation has been offered to all people diagnosed with HIV infection, regardless of their CD4^+^ status. The objectives of this procedure are to prevent viral resistance to cART and attain faster immune recovery^1^. This strategy has saved thousands of lives and is considered a unique initiative among middle-low-income countries^2^. With antiretroviral therapy (cART), most patients are under viremic control, with 60 to 90% efficacy^1,3^.

Several factors are related to the success of cART, including T CD4^+^ cell nadir, RNA HIV viral load at baseline, T-cell activation^4,5^, and other factors^6^. However, a characteristic of untreated HIV-1 infection is a sustained expansion in the number of circulating CD8^+^ T cells, which has been associated with increased CD8^+^ T cell activation, cycling, and turnover^7,8^. HIV infection is associated with immune activation, progressive CD4 T cell depletion, and immunodeficiency^9,10^. HIV can also contribute directly to the dysfunction of various organs, causing issues such as non-AIDS malignancies, cardiovascular events, renal and hepatic diseases, bone disorders, and neurocognitive impairment. These are the major causes of morbidity and mortality in the ART era^11,12^.

We hypothesized that, among individuals infected with HIV, treated with ART, and with CD4 counts ≥ 500 cells/mm^3^, the expansion of CD8+ T cells, reflected in a low CD4/CD8 ratio, may help identify those with persistent innate and adaptive immune activation and at greater risk of non-AIDS events. Since early ART initiation has been shown to reduce levels of T cell activation, we hypothesized that it might also accelerate the rate of CD4/CD8 ratio normalization and avoid the development of non-AIDS events.

This study aimed to evaluate the maintenance of the CD4^+^/CD8^+^ > 1 ratio during follow-up of patients undergoing early or delayed cART, to identify the risk factors for non-normalization of the CD4^+^/CD8^+^ ratio, and to verify the association of the index <1 with an AIDS event, death, and non-AIDS events, in a cohort of people living with HIV (PLHIV / AIDS) and starting cART^12^.

## MATERIALS AND METHODS

The pioneering outpatient service established in 1983 as a branch of the Clinical Immunology Service of the Hospital of Clinics of Sao Paulo included and followed up 1,100 patients infected with HIV-1, of whom 430 are currently under active follow-up. This study included treatment-naive patients who started cART from January 2012 to December 2015.

All treatment-naive patients were invited to take part in this prospective observational study. This analysis included 60 (13.9%) patients from the ADEE3002 cohort who had their viral load and CD4+ and CD8+ T cells measured three times per year. The inclusion criteria for the study were: being over 18 years old; having HIV infection (regardless of the time of infection); and never having been treated with ART. The study excluded pregnant women and patients with adverse events or any co-infection. The cART treatment was considered as early if it started immediately at the time of inclusion in the study and as late if it only started when patients had a CD4+ T cell count below 500 or opportunistic infections. Therefore, it is possible that some patients had a long infection time at the start of this study. In no case was treatment interrupted during follow-up. Follow-up consisted of observing the CD4+/CD8+ ratio and the response to treatment. The last CD4/CD8 ratio observed was considered the final outcome.

The project was approved by the Research Ethics Committee of the Institute of Tropical Medicine of Sao Paulo of the University of Sao Paulo under Nº CPE-IMT 000364. It was also approved by the Research Ethics Committee of the Faculty of Medicine of the University of Sao Paulo (CAPPesq) for analysis of research projects and at Plataforma Brasil, Nº 3.617.699.

The START trial was designed and conducted by the International Network for Strategic Initiatives in Global HIV Trials (INSIGHT). A description of the contributions of the study members is provided in Section 2 of the Supplementary Appendix, available with the full text of this article at NEJM.org. START ClinicalTrials.gov identifier: NCT00867048; START EudraCT Nº 2008-006439-12; INSIGHT NIAID grant Nº UM1 AI120197

### Phenotypic characterization of T cells by flow cytometry

The absolute counts of peripheral CD4^+^ and CD8^+^ T lymphocytes were calculated by flow cytometry using anti-CD3, anti-CD8, anti-CD45 and anti-CD4 monoclonal antibodies conjugated to the fluorochromes Fluorescein isothiocyanate (FITC), Phycoerythrin (PE), Peridinin Chlorophyll Protein (PerCP), and Allophycocyanin (APC), respectively. The acquisition was performed on a BD FACSCalibur cytometer (BD Biosciences, San Diego, CA, USA) using the MultiSet software (BD). The values (cells/μL) of these cell populations were calculated based on reference beads present in the tubes used for the tests (BD TruCount Tubes).

### Viral load quantification

HIV-1 RNA levels in plasma were quantified from peripheral blood samples, which were collected in EDTA and fractionated within four hours after collection by amplifying a gag region of the viral RNA, using real-time polymerase chain reaction (RT-PCR) with homogeneous real-time fluorescence detection (Abbott Real Time HIV-1^®^, Abbott Molecular, Inc. Des Plaines, USA), following the manufacturer’s instructions. The detection threshold of this test, which corresponds to the concentration of HIV-1 RNA detected with a probability of 95% or greater, is 40 copies/mL.

### Statistical analysis

The CD4^+^/CD8^+^ T cell ratio was analyzed as a dichotomous variable (<1 vs >1) according to the strategy employed at the start of the treatment, whether early or deferred. A log-rank test was performed for the comparison of survival curves. The T-test was used to compare the means of continuous variables, and the chi-square test was used to compare the values of categorical variables. Variables that reached a significance level of <0.2 in the univariate analysis were entered as independent variables in a multivariate logistic regression analysis. In this multivariate regression analysis, a p-value <0.05 was considered significant. To perform the statistical analyses, the GraphPad Prism 6 software (GraphPad Software, Boston, MA, USA) was used.

## RESULTS

Among the 430 patients, 60 were naive to antiretroviral therapy at the beginning of this study. They were followed up from January 2010 to February 2021. The early therapy group included 30 patients, who started cART from 2010 to 2012. The deferred therapy group included 30 patients who only started cART after their T CD4^+^ cell count decreased to less than 500 cells/mm3. In September 2015, all patients were encouraged to undergo treatment, but four patients in the deferred group declined and only started cART in 2019. TCD4^+^ and TCD8^+^ lymphocytes were counted every four months, and each patient underwent an average of 19 measurements during follow-up. The mean follow-up time was 6.7 years. The overall proportion of men in the study population was 80%, and women had a lower risk of not recovering the CD4^+^/CD8^+^ ratio. Higher CD4^+^ T lymphocyte counts at inclusion and lower CD8^+^ T lymphocyte nadir were associated with a ratio greater than 1 (p=0.02; p=0.01, respectively). Viral load, CD4^+^ nadir, CD8^+^ T cell count at inclusion, time of HIV infection, comorbidities at inclusion, and coinfections with hepatitis C and B were not associated with a worse CD4^+^/CD8^+^ ratio restoration (Table 1).

**Table 1:** Risk factors for the recovery of the CD4/CD8 cell ratio.

Characteristic	All 60 (100%)	Last CD4/CD8 ratio > 1 37 (61.6%)	Last CD4/CD8 ratio ≤ 1 23 (38.3%)	p-value
**Age,** mean, years, (±SD)	48.5 (8.6)	47.0 (8.8)	50.9 (8.1)	**0.04**
**Gender,** male, n (%)	48 (80.0)	27 (45.0)	21 (35.0)	0.08
female, n (%)	12 (20.0)	10 (16.6)	2 (3.3)	
**Viral load at inclusion**, mean, (±SD)	16,340 (35,386)	11,300 (18,756)	269,25 (51,343)	0.17
**Nadir CD4+T-cell count,** mean, (±SD)	479 (136)	473 (145)	492 (121)	0.6
**Nadir CD4+T-cell count before therapy,** mean, (±SD)	565 (209)	609 (244)	493 (106)	**0.01**
**CD4+ cell count at inclusion,** mean, (±SD)	791 (309)	855 (332)	692 (243)	**0.03**
**Nadir CD8+T-cell count,** mean, (±SD)	514 (185)	470 (173)	577 (187)	**0.02**
**CD8+ cell count at inclusion,** mm3, (±SD)	924 (360)	634 (408.2)	584 (187.0)	0.5
**Time of HIV infection,** years, (±SD)	10.7 (3.8)	10.8 (3.8)	10.6 (4.0)	0.8
**Therapy changes,** n (%)	4 (6.6)	3 (5.0)	1 (1.6)	0.28
**Mean time without ART,** years, (range)	5.0 (1–22)	4.7 (1–17)	5.0 (1–22)	0.76
**Comorbidities at inclusion,** n (%)	2 (3.3)	2 (3.3)	0	
**Coinfections,** n (%)	3 (5.0)	2 (3.3)	1 (1.6)	0.42
**Time to normalize the ratio after TARV,** mean years, (range)*		1.3 (0.3- 4.1)	23 Never normalized	
**Initial CD4 / CD8** ratio > 1, n (%)	18 (30.0)	17 (28.3)	1 (1.6)	**0.0003**
ratio ≤ 1, n (%)	42 (70.0)	20 (33.3)	22 (36.6)	

During follow-up, the incidence of AIDS was 18.3% (11/60), there was one death from AIDS, and half patients had some comorbidity. The comorbidities observed were metabolic syndromes (obesity, dyslipidemia, and diabetes), cardiovascular diseases (including arterial hypertension, myocardial infarction, and heart failure), and malignancies. The incidence of AIDS and non-AIDS events was not associated with a lower CD4^+^ / CD8^+^ ratio (Table 2).

**Table 2:** - Clinical events during follow-up among subjects infected with HIV.

Events, n, (%)	All 60 (100%)	Last CD4/CD8 ratio > 1 37 (66.6%)	Last CD4/CD8 ratio ≤1 23 (33.3%)	OR (CI)	p-value
AIDS	11 (18.3)	5 (8.3)	6 (10.0)	2.25 (0.48–10.7)	0.22
Death by AIDS	1 (1.6)	1 (1.6)	0		
Comorbidities	30 (50.0)	17 (28.3)	13 (21.6)	1.52 (0.47–4.98)	0.42
Metabolic Syndromes	27 (45.0)	15 (25.0)	12 (20.0)	0.62 (0.2–2)	0.37
Cardiovascular diseases	5 (8.3)	3 (5.0)	2 (3.3)	1.07 (0.98–10.2)	0.93
Malignancy	4 (6.6)	2 (3.3)	2 (3.3)	1.66 (0.11–24.4)	0.61

**Figure 1:**
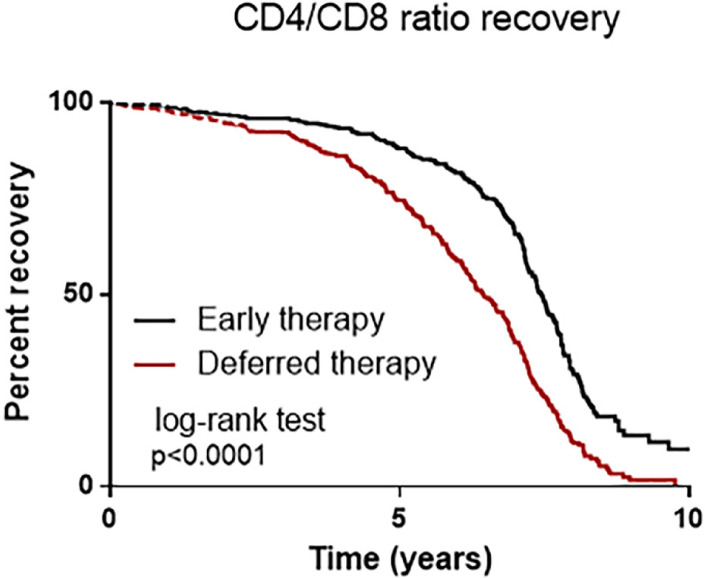
- T CD4+/CD8+ cell ratio progression along the study.

Patients who started follow-up with a ratio ≤1 had an 18-fold higher risk of not restoring the ratio (OR 19.5; CI 2.3–160.0; p=0.0004). In the multivariate analysis, starting treatment with a CD4^+^ / CD8^+^ ratio >1 was an indicator of maintaining a CD4^+^ / CD8^+^ above 1 over the years. The nadir CD4^+^ T-cell count before therapy and the nadir CD8^+^ T-cell count were not significant (Table 3). At the end of follow-up, four patients (6%) had HIV viral loads >40 copies/ml. The mean time without cART treatment during follow-up was five years for both groups. Three patients had coinfections, two patients were HCV-seropositive, one patient was HBV-seropositive, and no patients were positive for active CMV infection. The mean time needed to normalize the ratio after starting cART was 1.3 years (0.3–4.1 years), but 23 patients did not reach a higher-than-one (Figure 1).

**Table 3:** - Multivariate analysis including the significant variables in the univariate analysis. Only the initial CD4 ratio was associated with recovery at the end of follow-up.

Multivariate analysis	p-value
Nadir CD4^+^T-cell count before therapy	0.81
Nadir CD8^+^T-cell count	0.10
Initial CD4^+^/CD8^+^ ratio > 1	**0.0002**

In this model, only variables p<0.02 were included in this analysis; Multivariate analysis including the same variables that showed p<0.20 in the univariate analysis.

## DISCUSSION

The CD4^+^/CD8^+^ ratio has been proposed as a suitable marker of persistent immune dysfunction and of the occurrence of non-AIDS-related events in treated patients with HIV^13,14^. In this report, we described the evolution of the T CD4^+^/CD8^+^ cell ratio in long-term cART users from Sao Paulo, Brazil, and the association of the CD4^+^/CD8^+^ ratio with AIDS incidence. Despite the effectiveness of cART, the CD4^+^/CD8^+^ ratio does not always normalize in patients with HIV, especially in those with late start of treatment (deferred group) and low initial CD4^+^/CD8^+^ T-cell ratio. Early diagnosis should be encouraged so that patients are treated as soon as possible (promptly), favoring a more robust immunological reconstitution^15-17^. Although a low CD4^+^/CD8^+^ ratio is an indicator of CD4^+^ T cell lymphopenia and CD8^+^ T cell activation^18,19^, in this study, a lower result for this ratio was not associated with non-AIDS events, which may be associated with some independent comorbidities.

Generally, the patients in this study had a higher CD4+/CD8+ ratio compared to those in other studies, possibly due to early monitoring and the introduction of treatment as soon as the CD4^+^ T cell count fell below 500 cells/μL. Therefore, this cohort may not represent what happens in most centers that follow people living with HIV/AIDS. Longer follow-ups may reveal a higher incidence of non-AIDS events in patients with HIV who did not reach normalization of the ratio, strengthening the association of low CD4/CD8 ratios with non-AIDS morbidities^20-22^. The reversal of the CD4^+^/CD8^+^ ratio is considered an independent predictor of death in the general population, and the ratio physiologically decreases with aging^18^. CD8^+^ T cell expansion is linked to an increased risk of morbidity and mortality in patients with HIV-1 undergoing treatment^22-24^. The main factors associated with ratio restoration were the immediate start of cART and a CD4^+^ cell count greater than 500 cells/μL; whereas a higher baseline CD8^+^ cell count was negatively associated with ratio restoration^25,26^. The females seemed to present a lower risk of not restoring the ratio^24^.

Other studies linked a low CD4^+^/CD8^+^ ratio to neurocognitive disorders, suggesting that persistent T cell dysfunction contributes to memory decline^25^, but we have not yet assessed the cognitive function of all our patients to verify this association. In the general population, the association of CD4^+^/CD8^+^ ratio inversion with excess mortality affecting older people indicates that persistent inversion might be particularly relevant in older people diagnosed or living with HIV infection^27,28^. Therefore, it is important to investigate the impact of age on HIV diagnosis, particularly in patients with neurological impairment^29^.

The manipulation of this ratio could serve as a target for further therapeutic interventions against HIV, and the measurement of this ratio may serve as a surrogate for the HIV reservoir^30^. More knowledge is needed on how specific cART regimens, the simultaneous treatment of coinfections, and immunotherapy as a treatment for oncologic disorders impact the CD4^+^/CD8^+^ ratio.

## CONCLUSION

In conclusion, drug toxicity and a longer duration of antiretroviral therapy can affect the evolution of the CD4+/CD8+ ratio in the presence of comorbidities. Major CD4^+^/CD8^+^ ratio increases were observed during early therapy, and the maintenance of a ratio greater than one was found to be associated with a higher ratio at the beginning of treatment. Viral suppression and CD4 response will always remain important treatment goals in HIV management. However, if treatment success is defined solely by these parameters, we may fail to recognize certain risks encountered by today’s population with HIV. There is evidence supporting the fact that the CD4/CD8 ratio is a biomarker for assessing risks faced by the modern population with aviremic HIV. However, the impact of other immune stimuli—particularly non-HIV drugs and pathogens—on this ratio is mostly unknown.
